# Exotic species as models to understand biocultural adaptation: Challenges to mainstream views of human-nature relations

**DOI:** 10.1371/journal.pone.0196091

**Published:** 2018-04-30

**Authors:** Aline Dourado Sena Gama, Marcelo de Paula, Rafael Ricardo Vasconcelos da Silva, Washington Soares Ferreira, Patrícia Muniz de Medeiros

**Affiliations:** 1 Universidade Federal do Oeste da Bahia, Programa de Pós-Graduação em Ciências Ambientais, Estrada para o Barrocão, Barreiras, Bahia, Brazil; 2 Universidade Federal de Alagoas, Centro de Ciências Agrárias, Rio Largo, Alagoas, Brazil; 3 Universidade de Pernambuco, Campus Petrolina, Petrolina, Pernambuco, Brazil; Universidade Federal de Goias, BRAZIL

## Abstract

A central argument in the research on traditional knowledge, which persists in the scientific literature, is that the entrance of exotic plants in local medical systems is directly associated with acculturation. However, this logic has put an end for a long period to efforts to understand why such species have so successfully entered socio-ecological systems or even their real role in such systems. This study provides evidence that (1) in some socio-environmental contexts, exotic medicinal species usually confer greater adaptive advantages to local populations, and (2) despite their general importance, exotic species only excel in medical systems when cost-benefit ratio is favorable to them. Thus, in order to avoid the loss of knowledge about native plants and to ensure biocultural conservation, it is necessary to create strategies to amplify the advantages of these species.

## Introduction

Many efforts have been devoted to understanding how humans adapt to different socio-environmental contexts. Attempts have been made to understand the strategies developed by human groups to deal with, for example, arid environments [1,2], natural disasters [3], climate change [4,5], both now and in the past [6]. A set of theoretical scenarios has been proposed to understand these adaptations, based on evaluations of human decision making directed to the environment, such as economic scenarios, which presuppose that these decisions follow a logic of optimization of returns [7,8] to observe alternative scenarios other than the one presented.

Although many studies have attempted to understand the complexity of relationships between humans and their environments, it is still necessary to advance the understanding of the human behavior associated with these relationships [9, 8]. In this sense, empirical research on the factors that can model human behavior in their interactions with the environment is still needed, as well as on human perceptions about resources that can provide bases for decision-making [8], or even on social relations in a group that can affect the adoption of certain behaviors [2], among others. The present research proposes to assist bridging this gap by evaluating the costs and benefits of using environmental resources based on local perception in order to provide insight into some of the cognitive mechanisms that can shape human behavior in its adaptations to the environment. We chose medicinal plant use as the *corpus* of this research because (1) this is commonly a very important dominium of many socioecological systems, since healthcare is essential for human subsistence, (2) local medicinal systems are commonly diverse and heterogeneous, with several diseases to be treated and many plant species employed in their treatments. Such heterogeneity would allow the evaluation of common and distinct foraging behaviors among therapeutic indications.

### Cost-benefit as a metric for adaptation

The integration of human behavior into cost-benefit models presents major challenges, especially due to the high amount of theories employed in different lines of research [8]. Although some models based on the cost-benefit logic are criticized [10], their contribution to studies that seek to explain the evolution of biological [11, 12, 13] and socioecological systems [14, 15, 16] is remarkable. An increasing number of investigations into socioecological systems have been adopting some of these theories and adapting these models to themes and problems pertinent to human behavior in the use of natural resources and their adaptive strategies. Among the experiences that use the cost-benefit logic in the study of socioecological systems, the researches that adopt the optimal foraging theory (OFT) emerge.

OFT emerged in classical ecology referring to animal behavior and skills to obtain food with greater efficiency, that is, lower cost and greater benefit, aiming at the reproductive success of the species [17, 18]. Therefore, this ecological theory suggests, that the problem of energy distribution and optimization is analogous to cost-benefit analysis in economics, in which benefit is the energy return and costs are the energy and time required to secure future incomes. In human ecology, the optimal foraging theory was adapted to understand the human behavior in the search for resources [17, 19, 20]. One of the challenges of working with this theory in human ecology studies is the disparity between the quantitative and precise nature of the predictions and the qualitative nature of the empirical results [21]. This makes greater efforts necessary in the continuous methodological improvement to test this theory.

Regarding useful plants, most of the methods developed for the subject are related to food plants. These studies involve environmental and social aspects, considering the time of search and collection risks, the energy gain and the nutritive potential for the optimization of the forage [17, 19, 20, 22].

As in other evolution studies, our research is based on the premise that better cost-benefit relationships result in a greater adaptive advantage, which would partially guide human behavior in the use and management of natural resources. Thus, from the biocultural point of view, we believe that the evolutionary process follows the parameters highlighted by Albuquerque et al. [23], which are: (1) variation in characteristics of resources, especially in terms of availability; (2) competition between behaviors that exhibit the same cultural function and produce similar results; (3) selection of certain behaviors that generate a better cost-benefit ratio; (4) adaptation of a selected behavior as a result of the choices; (5) fixation of the most adapted mechanisms in the population, through social learning, thus allowing the system to accumulate information and evolve over time.

### Exotic species as a model for assessing biocultural evolution

Species that occur outside their natural biogeographic environment are considered exotic, being they dispersed intentionally or accidentally through human or dispersers action such as wind and animals [24]. Several exotic plant species only persist in certain localities because of their cultivation by humans, while others can be found spontaneously in disturbed environments or in agricultural fields, many of them with strong invading potential.

Due to their broad ecological and economic importance, exotic plants have been the subject of research in various areas of knowledge. In the case of socioecological systems, the appropriation of exotic plants by certain human populations has been viewed with concern by researchers of traditional knowledge. Such caveats to alien plants may be rooted in the idea that traditional knowledge and lifestyles need to be protected from external forces that can de-characterize them.

A central argument in research on traditional knowledge that persists in the scientific literature, is that the entrance of exotic plants is directly associated with acculturation. However, this way of thinking has, for a long period, put an end to efforts to understand why such species have so successfully entered socio-ecological systems, or even their real role in such systems. More recently, based on the idea that traditional knowledge is inherently adaptive [25], studies have sought to fill some of the gaps described above, most of which have been developed in the domain of medicinal plants. Considering the aspects that lead an exotic species to enter a medical system, the study of Bennett and Prance [26] suggested that these are settled primarily as food or ornamental and later people eventually identify its medicinal value. Thus, exotic species would be expected to be widely versatile (useful for multiple purposes), since such versatility would increase their chances of being recognized as medicinal. However, a study developed in the Brazilian semi-arid region indicated that most exotic plants have a specific medicinal purpose [27], which undermines this hypothesis.

Another proposition to explain the entrance of exotic species is that they enter the pharmacopoeias to diversify the repertoire of plants and enrich the pharmacological actions, since they present large amounts of secondary compounds or distinct types of secondary compounds from those found in native plants. Thus, the exotic species fill the gaps that are not filled by native plants [27, 28]. This hypothesis presented some favorable evidence, especially from the chemical point of view [27]. However, a systematic review performed for Brazil evidenced a high overlap of therapeutic niches (therapeutic functions) between native and exotic plants [29], which was confirmed by a later study in southeast Brazil [30]. Such overlap, associated with the presence of some gaps (therapeutic indications with absence or low use of native plants) leads to two possibilities: (1) entrance of exotic plants to fill gaps and subsequent dissipation into other therapeutic purposes already filled by native species, or (2) entrance of exotic species directly to compete with native ones and subsequent gaps formation through competitive exclusion of native plants [31].

The adaptive character of the entrance of exotic plants into local medical systems was developed theoretically by Medeiros [31]. The author outlines some possible adaptive advantages of such species, such as therapeutic efficacy, the lowest adverse effects, the most pleasant taste and the easiest acquisition.

Thus, although the replacement of native and exotic species can be considered negative from the point of view of cultural registration and bioprospecting, it is important to be cautious in attributing such judgments to the substitution process as a biocultural adaptation phenomenon [31]. It should be considered if, in the contexts of prominence of exotic plants, these species offer greater adaptive advantages, which would motivate people to use them. In this sense, the present study is the first to provide analytical tools to identify the potential of exotic species in a context of biocultural adaptation. Thus, it starts with the following hypotheses: 1) exotic species present adaptive advantages that justify their popularity, 2) the prominence of plant species in pharmacopoeias is explained by their cost-benefit relations, so that exotic species only excel when this relation is favorable to them, conferring them adaptive advantages.

## Materials and methods

### Study area

The study was developed in the rural community Morrão de Cima, in the municipality of São Desidério, Bahia state. The municipality of São Desidério has an economy based on farming and family farming.

The municipality covers a territorial area of 15.174,235 km^2^ [32]. The total population is of approximately 32.640 inhabitants [32]. The city of São Desidério is located in the west of Bahia and its temperature varies from 17°C to 37°C, with a drought period between May and September [33].

Morrão de Cima has 30 residences with approximately 75 residents among adults and children. The community does not have some public services, such as school and health center. However, a bus takes students to study in the urban area of São Desidério. The main economic activity of the community is family farming and animal husbandry.

Although the community does not have a health center, health agents from the urban area usually visit Morrão de Cima to offer dwellers basic health care. Local medical system is hybrid. People often consume allopathy, especially to treat more severe diseases, but all families use medicinal plants (alone or together with allopathy, in order to amplify chances for health improvement) and plants are still the main medicinal resource in the community. Reasons for medicinal plant importance in Morrão de Cima are (1) the cultural importance of such resources and (2) the high costs of allopathy, sometimes inaccessible to community members.

The vegetation surrounding the community is placed in the *Cerrado* dominium (Brazilian Savannah) and its phytophysiognomies. In the location, there are mountain ranges, plateaus (*chapadas*), and flat lands. Vegetation is composed of forests, grasslands and palm swamps (*veredas*).

### Ethics statement

Firstly, previous visits were made to the community in order to present the project. During this period, the project was submitted to the ethics committee of the Faculdade São Francisco de Barreiras, and it was approved for completion (CAAE 44962515.5.0000.5026). All participants were invited to participate the research and those who agreed also signed a free and clarified consent term, according to the resolution 196/96 of the National Health Council.

### Data collection

The interviews were developed in two stages. In the first stage we performed a semi-structured interview with 44 residents. Our goal was to interview all residents over 18 (48) However, one family chief refused to participate and three of them were not found (they worked outside the community and could only be found at night). These interviews were about socioeconomic information, as well as a free list of known medicinal plants, their use, the part used and the method of preparation. Later, in the second stage, four therapeutic indications were selected, which served as models for the pharmacopoeia.

Two diseases were selected in the group of lower severity (influenza and general inflammations) and two in the group of higher severity (cancer and high blood pressure). In each group were selected those diseases that presented the greatest number of species cited by at least two individuals in the first stage of the study (free list). Besides presenting the highest number of plant species cited for their treatment, community members are all familiar to these diseases, since influenza and general inflammations are common affections in the community and cancer and high blood pressure, although not spread, are part of people’s knowledge systems (all community members are close to someone with cancer or high pressure).

For each therapeutic indication, a checklist-interview was conducted [34], containing all the plants that were cited by more than one interviewee. These plants were presented to the interviewees, regardless of whether they had mentioned them or not in the semi-structured interview. Thus, the interviewees were asked to score, according to their perception, each plant considering: (1) the use they make of it, (2) efficiency, (3) taste, (4) availability and (5) adverse effects.

Interviewees assigned scores between 0 and 10 for each plant in each factor evaluated. In cases where the same plant was cited for different therapeutic indications or had more than one part used, they could present different scores. In order to complement the scoring exercise, the informant was asked to rank the scored plants. For the presentation of the plants, letters with the photo and popular name of the plant were used, and these were mixed at each interval to avoid biasing the scoring and/or ranking.

A total of 31 people participated in the second stage, considering that 13 of the participants from the previous stage were not willing to participate or were not found in their residences.

The key informants in the research conducted the guided tour to collect the cited plants [34]. These plants were herborized and deposited in the herbarium of the Federal University of Western Bahia (UFOB). Then they were identified and classified according to the specialized literature and websites such as the list of species of Flora do Brasil (http://floradobrasil.jbrj.gov.br/). For the classification of native and exotic species, their origin for the Cerrado was considered.

### Data analysis

Two measurement methodologies were considered for data analysis: scores and ranking. For this, it was necessary to make an inverse interpretation of the magnitudes by ranking in relation to the scores. For instance, we considered that the value ten corresponds to the highest score a plant could get (but interviewees were free to give any grade for a given species), while in relation to the ranking, the value one (first place) was attributed for the most outstanding plant in a given parameter. The analysis is divided into three inseparable parts: (1) Descriptive analysis; (2) Inferential analysis via statistical test of comparison between exotic and native origins for all symptoms and both methodologies; (3) Regression analysis to detect the significant variables that remain in the model.

Thus, we considered as independent variables the average scores for the perception of informants about efficiency, taste, availability and adverse effects, to explain the use that is the dependent variable. The efficiency and taste factors are considered benefits, while availability and adverse effects are considered costs. Thus, the variable perceived efficiency corresponds to the best product to treat the disease. For the perceived availability variable we agreed that the further the species is, the higher the energy expenditure for its acquisition, that means, the higher the cost. The most pleasant taste induces the use of medicinal plants, which is considered a benefit. In contrast, the adverse effects may inhibit the use of plant species, which can be considered a cost.

In order to test the first hypothesis (exotic species present adaptive advantages that justify their popularity), an inferential analysis was performed using a Student-T Test with a significance level of 5%, for comparison between exotic and native origins regarding the perception of informants on the factors: efficiency, taste, availability and adverse effects. This analysis was performed for all symptoms and both methodologies (scores and ranking). The hypothesis is confirmed if the exotic species excel in more variables (efficiency, taste, availability and adverse effects) than the native ones.

In the second hypothesis (the prominence of plant species in pharmacopoeias is explained by their cost-benefit relations, so that exotic species only excel when this relation is favorable to them) a multiple linear regression model was used to identify the variables that best explain the use, considering four levels of configurations for each methodology (Table 1). The hypothesis is confirmed in the case of at least one cost variable and one benefit variable remain in the models.

**Table 1:** Configuration levels for regression analyses used to identify the variables that explain medicinal plant differential use in the rural community of Morrão de Cima, municipality of São Desidério, Northeastern Brazil.

Configuration		Method	
		Grades	Ranking
First level	All symptoms together	Both origins	Both origins
Second level	All symptoms together	Exotic	Exotic
		Native	Native
Third level	Cancer Influenza Inflammation High pressure	Both origins	Both origins
Fourth level	Cancer	Exotic	Exotic
		Native	Native
	Influenza	Exotic	Exotic
		Native	Native
	Inflammation	Exotic	Exotic
		Native	Native
	High pressure	Exotic	Exotic
		Native	Native

For all regression analyses, a selection of variables was performed using the Stepwise technique, hence only the variables that provide the best fit remain in the model to explain the use. The regression analysis was based on the results obtained from the four levels of configurations, considering both methodologies, totalizing 30 adjustments of models, according to Table 1.

## Results

### General aspects

Considering the four therapeutic indications that served as model for the present study (influenza, inflammation, high blood pressure and cancer), a total of 43 plants were cited by at least two informants, some of which are used for more than one therapeutic indication (Table 2). In some cases, more than one part of the plant has been mentioned to treat the same disease.

**Table 2:** Plants used by at least two interviewees for cancer, high pressure, influenza and inflammation and their mean values (grades and rankings). Data from an ethnobotanical inventory performed in the rural community of Morrão de Cima, municipality of São Desidério, Northeastern Brazil. BRBA: voucher number for the herbarium of *Universidade Federal do Oeste da Bahia*. Origin (native and exotic) for the Brazilian *Cerrado*.

Species	Popular name	Used part	Origin	Average grades for use	Average ranking for use	BRBA
**CANCER**						
*Aloe vera* (L.) Burm.f.	Babosa	Leaf	Exotic	7.52	2.61	6713
*Annona muricata* L.	Graviola	Fruit	Exotic	6.69	3.14	6702
*Croton* cf. *Sebastiana*	Velame	Leaf	Native	3.33	5.67	6741
*Euphorbia tirucalli* L.	Avelós	Exudates	Exotic	7.48	2.78	6740
*Hancornia speciosa* Gomes	Mangabá	Exudates	Native	6.91	3.61	6705
*Undetermined*	Bacuparé	Seed	Native	1.91	6.5	6856
*Jacaranda brasiliana* (Lam.) Pers.	Carobinha	Leaf	Native	3	6	6719
*Morinda citrifolia* L.	None	Leaf	Exotic	4.64	4.39	6815
*Morinda citrifolia* L.	None	Fruit	Exotic	7.27	2.83	6815
**INFLUENZA**						
*Allium sativum* L.	Alho	Bulb	Exotic	7.71	6.68	6689
*Anadenanthera colubrina* (Vell.) Brenan	Angico	Bark	Native	3.79	14.48	6744
*Anadenanthera colubrina* (Vell.) Brenan	Angico	Exudates	Native	3.97	12.86	6744
*Bowdichia virgilioides* Kunth	Sucupira	Bark	Native	3	15.67	6745
*Bowdichia virgilioides* Kunth	Sucupira	Seed	Native	7.1	9.23	6745
*Brosimum gaudichaudii* Trécul	Bureré	Bark	Native	1.73	18.07	6792
*Brosimum gaudichaudii* Trécul	Bureré	Exudates	Native	1.2	19.87	6792
*Cajanus cajan* (L.) Millsp.	Andu	Leaf	Exotic	2.39	16.19	6746
*Caryocar brasiliense* A.St. -Hil.	Pequí	Fruit	Native	6.16	11.35	6729
*Citrus limon* (L.) Burm. f.	Limão	Fruit	Exotic	8.81	5	6816
*Copaifera luetzelburgii* Harms	Pau d'óleo	Exudates	Native	2.94	15.06	6750
*Cymbopogon citratus* (DC.) Stapf.	Capim santo	Leaf	Exotic	5.74	9.61	6806
*Undetermined*	Carapiá	Leaf	Native	1.7	18.11	6836
*Undetermined*	Carapiá	Raíz	Native	6	11.56	6836
*Undetermined*	Quina	Bark	Native	3.33	15.75	6853
*Lipia alba* (Mill.) N.E.Br.	Erva Cidreira	Leaf	Exotic	6.16	10.55	6826
*Malpighia emarginata* DC.	Acerola	Fruit	Exotic	6	8.48	6785
*Mangifera indica* L.	Manga	Leaf	Exotic	1.87	16.52	6695
*Mauritia flexuosa* L.f.	Buriti	Fruit	Native	4.33	13.53	6711
*Mentha* sp.	Hortelã miúdo	Leaf	Exotic	6.03	10.84	6771
*Mentha pulegium* L.	Puejo	Leaf	Exotic	6.23	10.57	6709
*Myracrodruon urundeuva* Allemão	Aroeira	Bark	Native	2.5	15.47	6696
*Ocimum basilicum* L.	Manjericão	Leaf	Exotic	5.23	12.32	6773
*Ocimum gratissimum* L.	Alfavaca	Leaf	Exotic	7.97	7.35	6774
*Periandra mediterranea* (Vell.) Taub.	Alcançu	Bark	Native	3.21	18.06	6759
*Periandra mediterranea* (Vell.) Taub.	Alcançu	Raíz	Native	5.75	14.19	6759
*Petiveria alliacea* L.	Tipí	Leaf	Exotic	2.89	16.25	6804
*Petiveria alliacea* L.	Tipí	Raíz	Exotic	4.71	13.32	6804
*Plectranthus amboinicus* (Lour.) Spreng.	Hortelã grosso	Leaf	Exotic	4.45	12.77	6775
*Protium heptaphyllum* (Aibl.) Marchand	Amescla	Bark	Native	3.21	16.79	6724
*Protium heptaphyllum* (Aibl.) Marchand	Amescla	Exudates	Native	5.42	12.46	6724
*Ruta graveolens* L.	Arruda	Leaf	Exotic	5.23	11.13	6820
**INFLAMMATION**						
*Aloe vera* (L.) Burm.f.	Babosa	Leaf	Exotic	5.48	7.61	6713
*Anacardium humile* A.St.-Hil.	Cajuí	Bark	Native	2.71	11.19	6692
*Anacardium occidentale* L.	Cajú	Bark	Native	3.71	9.77	6693
*Anadenanthera colubrina* (Vell.) Brenan	Angico	Bark	Native	4.61	9.14	6744
*Bowdichia virgilioides* Kunth	Sucupira	Bark	Native	5.17	9.37	6745
*Bowdichia virgilioides* Kunth	Sucupira	Seed	Native	5.97	7.13	6745
*Bryophyllum pinnatum* (Lam.) Oken	Leaf Santa	Leaf	Exotic	2.91	11.41	6735
*Calliandra dysantha* Benth.	Paratudo	Root	Native	4.41	8.59	6747
*Chenopodium ambrosioides* L.	Mastruz	Leaf	Exotic	8.94	4.03	6691
*Croton* cf. *Sebastiana*	Velame	Leaf	Native	8.55	3.09	6741
*Dimophandra gardineriana* Tur.	Barbatimão	Bark	Native	6.7	6.82	6751
*Dimophandra gardineriana* Tur.	Barbatimão	Fruit	Native	4.52	8.54	6751
*Euphorbia tirucalli* L.	Avelós	Exudates	Exotic	2.25	11.46	6740
*Gossypium hirsutum* L.	Algodão	Leaf	Exotic	7.16	5.42	6787
*Hymenaea stigonocarpa* Mart. ex Hayne	Jatobá	Bark	Native	4.84	7.97	6752
*Hymenaea stigonocarpa* Mart. ex Hayne	Jatobá	Exudates	Native	4.77	7.42	6752
*Undetermined*	Quina	Bark	Native	3.89	10.11	6853
*Lafoensia pacari* A.St.-Hil.	Pacarí	Bark	Native	8.57	3.96	6783
*Morinda citrifolia* L.	None	Leaf	Exotic	0.74	12.43	6815
*Morinda citrifolia* L.	None	Fruit	Exotic	3.44	10.5	6815
*Myracrodruon urundeuva* Allemão	Aroeira	Bark	Native	5	9.54	6696
*Ouratea hexasperma* Baill.	Cabelo de nego	Bark	Native	5.24	9.59	6800
*Plantago major* L.	Transagem	Leaf	Exotic	3.94	11.33	6805
*Plectranthus amboinicus* (Lour.) Spreng.	Hortelã grosso	Leaf	Exotic	4.42	9.39	6775
*Punica granatum* L.	Romã	Fruit	Exotic	7.35	5.93	6784
*Terminallia fagifolia* Mart. & Zucc	Muçambê	Bark	Native	7.82	8.71	6733
**HIGH PRESSURE**						
*Averrhoa carambola* L.	Carambola	Leaf	Exotic	2.41	5.69	6801
*Averrhoa carambola* L.	Carambola	Fruit	Exotic	3.76	4.72	6801
*Cymbopogon citratus* (DC.) Stapf.	Capim santo	Leaf	Exotic	6.65	3.77	6806
*Eugenia dysenterica* Mart ex DC.	Cagaita	Bark	Native	2.42	5.5	6795
*Eugenia dysenterica* Mart ex DC.	Cagaita	Leaf	Native	3.38	5.15	6795
*Eugenia dysenterica* Mart ex DC.	Cagaita	Fruit	Native	3.23	4.85	6795
*Undetermined*	Budim	Root	Native	4.14	4.64	6861
*Lipia alba* (Mill.) N.E.Br.	Erva Cidreira	Leaf	Exotic	8	3.03	6826
*Passiflora edulis* Sims	Maracujá	Fruit	Native	8.16	2.32	6802
*Rosmarinus officinalis* L.	Alecrim	Leaf	Exotic	5.03	3.81	6777
*Spondias mombin* L.	Siriguela	Leaf	Exotic	7.55	3.45	6698

Both native and exotic plants were cited for the four therapeutic indications. Only the indication inflammation obtained more citations of native plants, so that, for the other diseases the exotic species excelled in quantity of species and in number of citations. Influenza had 25 plants selected, of which 11 native and 14 exotic; inflammation had 22 plants selected, 13 native and 9 exotic; high blood pressure had 8 plants selected, 3 native and 4 exotic; and cancer had 8 plants selected, 4 for each origin. Among the prominent exotic species, the great majority is cultivated.

When considering average scores and rankings of use, exotic species excel with higher average scores and lower average rankings (Table 2). The species with the highest average scores were *Aloe vera* (L.) Burm.f., *Euphorbia tirucalli* L. and *Morinda citrifolia* L. for cancer; *Citrus limon* (L.) Burm. f., *Ocimum gratissimum* L. and *Allium sativum* L. for influenza; *Chenopodium ambrosioides* L., *Lafoensia pacari* A.St.-Hil. and *Croton* cf. *sebastiana* for general inflammation, and *Passiflora edulis* Sims, *Lipia alba* (Mill.) N.E.Br. and *Spondias mombin* L. for high blood pressure (Table 2).

Among the most outstanding species, all those for cancer and influenza are native to the old world (*A*. *vera*, *M citrifolia*, *C*. *limon* and *A*. *sativum* are native to Asia, while *E*.*tirucalli*, and *O*. *gratissimum* are native to both Asia and Africa). Interestingly, all outstanding species for general inflammation and high blood pressure are native to America (*C*. *ambrosioides*, *L*. *alba* and *S*. *mombin* are native to America but not to the *Cerrado* and *L*. *pacari*, *C*. cf. *sebastiania* and *P*. *edulis* are native to the *Cerrado*).

### Exotic species present adaptive advantages that justify their popularity

Hypothesis 1 was confirmed, since the exotic plants excelled for all the variables tested. These results, with some exceptions, were observed both evaluating all symptoms together and each symptom individually (Tables 3 and 4).

**Table 3:** Descriptive statistics for the database of plants used by residents of the community of Morrão de Cima, municipality of São Desidério, Northeastern Brazil. Data for the Symptoms Cancer, Influenza, Inflammation and High pressure. Higher mean values mean higher use, higher efficiency, better taste and more side effects.

**Symptoms**	**Exotic species**							
	**Grades**				**Ranking**			
**Cancer**	**N° of observations**	**Mean values**	**Standard deviation**	**Variation coefficient**	**N° of observations**	**Mean values**	**Standard deviation**	**Variation coefficient**
Use	141	6.7	4.01	59.8%	138	3.1	2.01	64.3%
Efficiency	141	6.5	4.09	62.9%	138	2.8	1.78	62.5%
Availability	141	7.4	3.48	46.9%	138	3.2	2.74	85.8%
Taste	141	3.4	3.70	110.4%	138	3.6	2.22	61.8%
Side effect	141	3.8	4.53	119.4%	138	2.3	1.45	62.5%
**Influenza**	**N° of observations**	**Mean values**	**Standard deviation**	**Variation coefficient**	**N° of observations**	**Mean values**	**Standard deviation**	**Variation coefficient**
Use	472	5.3	4.02	76.0%	460	11.5	7.52	65.2%
Efficiency	472	5.5	4.02	73.0%	460	11.8	7.58	64.4%
Availability	472	7.6	3.26	43.1%	460	10.4	8.26	79.6%
Taste	472	5.7	4.05	70.8%	460	10.9	8.24	75.4%
Side effect	472	1.2	2.74	232.8%	460	9.5	5.27	55.5%
**Inflammation**	**N° of observations**	**Mean values**	**Standard deviation**	**Variation coefficient**	**N° of observations**	**Mean values**	**Standard deviation**	**Variation coefficient**
Use	272	4.9	4.34	88.4%	266	9.5	6.74	70.7%
Efficiency	272	5.3	4.36	82.4%	266	9.7	6.52	67.2%
Availability	272	6.9	3.73	54.3%	266	8.1	6.81	84.5%
Taste	272	4.0	3.92	97.4%	266	9.2	6.98	76.0%
Side effect	272	2.6	4.00	154.3%	266	7.8	6.03	77.5%
**High pressure**	**N° of observations**	**Mean values**	**Standard deviation**	**Variation coefficient**	**N° of observations**	**Mean values**	**Standard deviation**	**Variation coefficient**
Use	182	5.6	4.32	76.8%	178	4.2	2.57	61.4%
Efficiency	182	5.4	4.26	78.5%	178	4.4	2.46	56.0%
Availability	182	7.0	3.72	53.0%	178	3.8	2.56	67.5%
Taste	182	6.2	4.08	65.9%	178	4.4	2.70	61.2%
Side effect	182	1.6	3.21	196.3%	178	2.8	2.21	80.3%
**Symptoms**	**Native species**							
	**Grades**				**Ranking**			
**Cancer**	**N° of observations**	**Mean values**	**Standard deviation**	**Variation coefficient**	**N° of observations**	**Mean values**	**Standard deviation**	**Variation coefficient**
Use	50	4.6	4.37	95.4%	47	5.0	2.16	43.1%
Efficiency	50	3.8	4.41	116.8%	47	4.3	2.18	50.4%
Availability	50	5.2	3.13	60.7%	47	5.8	2.47	42.4%
Taste	50	3.7	3.80	103.8%	47	3.9	2.50	64.1%
Side effect	50	1.5	3.01	195.6%	47	3.1	1.70	54.6%
**Influenza**	**N° of observations**	**Mean values**	**Standard deviation**	**Variation coefficient**	**N° of observations**	**Mean values**	**Standard deviation**	**Variation coefficient**
Use	434	4.0	3.94	99.5%	434	14.9	7.37	49.6%
Efficiency	434	4.6	4.21	90.6%	434	13.7	7.81	57.1%
Availability	434	5.5	3.34	61.2%	434	18.5	9.20	49.7%
Taste	434	3.6	3.50	98.5%	434	16.9	7.61	45.1%
Side effect	434	3.6	4.19	116.1%	434	7.7	5.51	71.2%
**Inflammation**	**N° of observations**	**Mean values**	**Standard deviation**	**Variation coefficient**	**N° of observations**	**Mean values**	**Standard deviation**	**Variation coefficient**
Use	426	5.1	4.31	84.7%	428	8.7	5.83	67.3%
Efficiency	426	5.4	4.23	78.5%	428	9.4	5.83	61.9%
Availability	426	5.6	3.47	62.2%	428	11.7	6.48	55.2%
Taste	426	3.8	3.44	91.4%	428	9.4	5.50	58.2%
Side effect	426	4.1	4.24	103.8%	428	6.7	4.95	73.6%
**High pressure**	**N° of observations**	**Mean values**	**Standard deviation**	**Variation coefficient**	**N° of observations**	**Mean values**	**Standard deviation**	**Variation coefficient**
Use	131	4.4	4.53	102.6%	130	4.7	2.68	57.5%
Efficiency	131	4.2	4.40	105.2%	130	4.9	2.69	55.2%
Availability	131	4.6	3.77	82.7%	130	6.1	3.16	51.8%
Taste	131	5.2	4.26	82.0%	130	5.2	2.95	57.1%
Side effect	131	2.2	3.76	167.7%	130	2.5	2.12	85.5%

**Table 4:** *Student’s t*-test results for comparisons between native and exotic plant species regarding average grades and rankings for ‘use’, ‘efficiency’, ‘availability’ and ‘taste’ in the community of Morrão de Cima, municipality of São Desidério, Western Brazil. Higher mean values mean higher use, higher efficiency, better taste and more side effects. P-values equal to or lower than 0.05 are considered statistically significant. Df = degrees of freedom.

Symptoms	Variables	Grades				Ranking			
		t-test	df	p-value	Highest mean	t-test	df	p-value	Highest mean
All symptoms together	Use	5.091	2104	<0.001	Exotic	-6.160	2076	<0.001	Native
	Efficiency	3.889	2101	<0.001	Exotic	-5.157	2078	<0.001	Native
	Availability	12.507	2106	<0.001	Exotic	-16.863	2011	<0.001	Native
	Taste	7.151	2086	<0.001	Exotic	-10.228	2072	<0.001	Native
	Side effect	-9.291	2039	<0.001	Native	2.090	2066	0.037	Exotic
Cancer	Use	3.032	80	0.003	Exotic	-5.241	75	<0.001	Native
	Efficiency	3.811	81	<0.001	Exotic	-4.202	68	<0.001	Native
	Availability	4.264	95	<0.001	Exotic	-6.944	69	<0.001	Native
	Taste	-0.492	84	0.624	**-**	-0.730	72	0.468	**-**
	Side effect	3.943	130	<0.001	Exotic	-2.847	70	0.006	Native
Influenza	Use	5.039	900	<0.001	Exotic	-6.704	891	<0.001	Native
	Efficiency	3.124	889	0.002	Exotic	-3.707	885	<0.001	Native
	Availability	9.588	893	<0.001	Exotic	-13.894	868	<0.001	Native
	Taste	8.644	900	<0.001	Exotic	-11.216	892	<0.001	Native
	Side effect	-10.239	737	<0.001	Native	4.861	883	<0.001	Exotic
Inflammation	Use	-0.514	574	0.607	**-**	1.761	501	0.079	**-**
	Efficiency	-0.304	565	0.762	**-**	0.560	515	0.576	**-**
	Availability	4.598	547	<0.001	Exotic	-7.059	541	<0.001	Native
	Taste	0.914	522	0.361	-	-0.506	466	0.613	**-**
	Side effect	-4.703	602	<0.001	Native	2.358	482	0.019	Exotic
High pressure	Use	2.358	272	0.019	Exotic	-1.523	272	0.129	**-**
	Efficiency	2.502	275	0.013	Exotic	-1.633	263	0.104	**-**
	Availability	5.692	278	<0.001	Exotic	-6.832	242	<0.001	Native
	Taste	2.061	273	0.040	Exotic	-2.290	263	0.023	Native
	Side effect	-1.494	252	0.136	**-**	1.075	284	0.283	**-**

The exotic species presented a higher average score for the variables efficiency, availability and taste, suggesting that, according to the local perception, exotic species are more efficient, more available and with better taste. The adverse effect variable presented a higher average score for the native plants, which indicates that, according to the local perception, the exotic species present less harmful effects (Tables 3 and 4).

Considering the ranking with inverse values of the scores, the exotic species obtained lower averages in relation to the native plants, except for the adverse effect variable, with higher average ranking of the exotic ones. Exotic species, in general, also obtained a lower coefficient of variation (Table 3), that is, lower variability.

### Exotic species only excel when they present adaptive advantages

Hypothesis 2 was also confirmed. Table 5, in the first configuration level, shows that all the cost and benefit variables remain in the model to explain the use of native and exotic plants, both in the measurement methodology by scores and ranking. The hypothesis is confirmed at all configuration levels, since at least one benefit variable and one cost variable remain in the models to explain the use of plant species (except for cancer at level 3, for which only the benefit variables remained).

**Table 5:** Regression coefficients after Stepwise variable selection for predicting the variable ‘use’ with both grades and ranking methods. Data for the community of Morrão de Cima, municipality of São Desidério, Western Brazil. RMS = Residual mean square.

**Measure with grades**								
**Level description**			**Intercept**	**Variable**				**RMS**
				**Efficiency**	**Availability**	**Taste**	**Side effect**	
First level	All symptoms and both origins		0.015	0.750***	0.041**	0.158***	0.030*	4.404
Second level	All symptoms	Exotic	-0.033	0.762***	0.067**	0.133***	0.034^.^	4.134
		Native	0.083	0.737***	-	0.185***	0.039*	4.648
Third level	Cancer	Both origins	0.922***	0.856***	-	0.082*	-	3.203
	Influenza		-0.110	0.680***	0.081***	0.162***	-	4.953
	Inflammation		-0.046	0.702***	-	0.280***	0.064***	4.142
	High pressure		0.142	0.901***	0.074*	-	0.059*	2.555
Fourth level	Cancer	Exotic	0.208	0.828*	0.123**	0.065^.^	-	2.280
		Native	2.124**	0.829***	-0.323**	0.198*	0.172	5.011
	Influenza	Exotic	-0.079	0.680***	0.082*	0.175***	-	4.945
		Native	-0.023	0.692***	0.061^.^	0.120**	-	4.953
	Inflammation	Exotic	0.024	0.674***	-	0.299***	0.046	4.062
		Native	0.093	0.727***	-0.055^.^	0.284***	0.076**	4.198
	High pressure	Exotic	0.071	0.906***	0.078*	-	0.052	2.296
		Native	0.126	0.883***	-	0.090*	0.059	2.951
**Measure with rankings**								
**Level description**			**Intercept**	**Variable**				**RMS**
				**Efficiency**	**Availability**	**Taste**	**Side effect**	
First level	All symptoms and both origins		0.693***	0.512***	0.083***	0.283***	0.041*	20.298
Second level	All symptoms	Exotic	0.741**	0.549***	0.135***	0.242***	-	18.661
		Native	0.840**	0.481***	0.058**	0.337***	-	21.693
Third level	Cancer	Both origins	0.032	0.725***	0.125**	0.099*	0.162*	1.525
	Influenza		2.332***	0.459***	0.081***	0.278***	-	29.906
	Inflammation		1.126**	0.578***	-	0.252***	-	19.579
	High pressure		0.397^.^	0.702***	-	0.111**	0.091*	2.704
Fourth level	Cancer	Exotic	-0.133	0.772***	-	0.149**	0.228**	1.429
		Native	1.106*	0.689***	0.158^.^	-	-	1.712
	Influenza	Exotic	1.953***	0.493***	0.118***	0.234***	-	29.263
		Native	2.884***	0.425***	0.043	0.319***	-	30.425
	Inflammation	Exotic	0.456	0.575***	0.151***	0.249***	-	18.954
		Native	1.338**	0.543***	-	0.233***	-	19.200
	High pressure	Exotic	0.695**	0.797***	-	-	-	2.807
		Native	-0.007	0.602***	0.112*	0.146*	0.116^.^	2.493

***p<0.001;

**p<0.01;

*p<0.05;

^.^p<0.1.

No signs indicate non-significant p-values.

The efficiency variable remains at all levels of configurations in both methodologies to explain species use. Thus, efficiency explains the use of plants regardless of origin, which shows the importance of this variable. Considering the ranking measurement, the side effect does not explain the use in most models. However, for the scores methodology, this variable explains the use in most cases.

When comparing the methodologies of scores and ranking, it is noticed that the scores present the best statistical adjustment, since they have the smallest residual mean square (RMS). Thus, the explanatory power of the models with scores is larger than those of the models with ranking.

## Discussion

Our results indicate that, in general, there is a tendency for exotic plants to have adaptive advantages over native ones. However, not all exotic species present such advantages and this represents a central point of the research. This is because it is not enough to be exotic to get prominence in the medical system. Our predictive models indicated that, regardless of their origin, only those species that offer adaptive advantages (lower costs and greater benefits) stand out for their use. Such observations contribute to the complexification of ideas that native species are replaced by exotic species simply by market access forces or the influence of external cultures. Thus, we demonstrate that "exotic by exotic" is not sufficient to ensure establishment and popularity in a local medical system.

From the point of view of biocultural evolution, considering the dynamics of plant selection for medicinal use in socioecological systems, the data suggest for the first time that the entrance of exotic species into human groups may be associated with the adaptive advantages these species offer when compared to native plants. This may also help to explain why exotic plants are found in medicinal use for various human groups [26].

Several authors have sought to explain the mechanisms that are involved in the selection of medicinal plants [35, 36], whether at the entrance of a new medicinal plant [35] or in choosing a particular plant for use over another [37]. Observing from our findings that the use of native and exotic plants was explained by perceived cost and benefit characteristics in medicinal use, it is likely that these characteristics together provide some of the mechanisms that affect the selection process of medicinal plants. For this idea to gain more strength, more studies need be conducted with human groups in different environments.

To what concerns the biogeographical origin of the most outstanding medicinal plant species, the fact that they come from both old and new world indicates that there was no single route that determined exotic species importance. The lack of unique routes for the arrival outstanding medicinal species may signalize that cost and benefit is more important than biogeographical influence when it comes to medicinal plant importance.

Among the outstanding species identified in the study, most are cultivated. Regarding the adaptive advantages of the cultivated exotic species, we believe that the process of domestication by which many of these plants were submitted can explain much of this. Therefore, a species whose evolution has been shaped by human management is more likely to present more advantages to humanity than a species that was not.

Thus, it is possible that the domestication process has contributed to amplifying the concentration of certain bioactive compounds in some exotic cultivated species, as well as reducing the concentration of compounds responsible for unpleasant taste or adverse effects. However, these factors would not always act together, since it is common for the same bioactive compound to be responsible for an unpleasant taste or for adverse effects [38]. Still, the propensity to cultivation may amplify the availability of the species, which would explain the importance of part of the exotic species (cultivated) on this factor.

However, it is necessary to emphasize that there are very few studies that are dedicated to understanding the effects of domestication on medicinal plants, most of them dedicated to food species [39, 40, 41]. Despite this paucity of studies, some researches have already challenged the idea that different therapeutic effects are only caused by environmental conditions and the habitats where medicinal plants are grown or collected, in order to demonstrate that genetic differentiation between plant populations can be responsible for different concentrations of bioactive compounds [42, 43, 44].

From our data, availability explained the use of medicinal plants, both native and exotic. This finding disagrees with some works in the literature that show that the availability does not explain the importance of a plant in the medicinal use for several human groups. Gonçalves et al. [45], for instance, conducted a meta-analysis on the role of availability on the use of plants in several studies and observed that local availability of plants does not tend to explain their medicinal use. However, it is important to consider that these works evaluated the availability of plants through phytosociological parameters and our work started from the availability perceived by the people. It is possible to suggest that, when evaluating the perceived availability, we may be capturing some information that the availability measured by other parameters is not registering. For example, some plants may present a high availability in a fragment of local vegetation, but this fragment is distant and of hard accessibility to people in the community. This fact may lead to changes in the results of the effect of availability when it does not consider local perception. Furthermore, the studies evaluated in the meta-analysis [45] only considered the woody species available in forest areas, and exotic species may be responsible for strengthening the relation between use and availability.

Although all cost and benefit variables significantly influence medicinal plant importance, we found through informal interviews with some community members that availability is perceived as the most important factor that leads to a higher use of exotic species. One of our interviewees, for example, said that “it is hard for us to collect medicine from the woods. Sometimes we want to make a medicine and the [exotic] plant is already there, so we end up using what is close to our homes”. According to some community members, native species are only used when they are easily found close to people’s residence.

Some contexts can amplify the role of species availability in diminishing native medicinal plant use. First, some communities in NE Brazil are facing a strong emigration process, since young adults are moving to urban areas in order to find better jobs and improve their life quality (see, for example, Sieber et al. [46]). Therefore, such communities have most of their adult population composed of old adults, many of them with physical limitations to access forest areas, especially when those areas are far from the households. This scenario was responsible for the abandonment of agricultural areas and the recovery of forest environments in some communities from NE Brazil [46] and it possibly contributed to decreasing native medicinal plant harvesting in Morrão de Cima.

Second, people’s relationship with forest areas is changing in some Brazilian rural communities due to current restrictions in resource use. Wood harvesting for example, is becoming a challenge to local populations since people fear punishment from regulatory environmental agencies (such as IBAMA, a federal environmental agency). It is likely that when people make frequent incursions into forest areas to get woody products (e.g. firewood for their daily activities), chances of medicinal plant collection increase (people are already in the forest and may take the chance to collect additional resources). Therefore, we believe that such decrease in forest incursions to get woody resources may have diminished native medicinal plant collection.

As observed, the prominence of exotic species in the studied context is strongly associated with the adaptive advantages they presented in comparison to the native species. However, in other contexts, native species may excel in terms of greater adaptive advantage. Some studies attribute the prominence of natives to factors such as greater availability, seasonality, efficiency in the treatment of some diseases and aggregated cultural value [28, 47]. It should be added that, in addition to the mentioned factors, the limitations of certain environments to the cultivation of exotic species can also represent a decisive restrictive force for the entrance of exotic plants into these pharmacopoeias. In semi-arid regions in northeastern Brazil, for instance, several studies have demonstrated a predominance of native species in local pharmacopoeias [28, 48]. This may be associated with environmental adversities (especially water) in this region, which make it difficult to grow exotic species widely used and used in other parts of the world.

The findings of this study associated to contributions from the literature contribute, albeit in an incipient way, to demystify the idea that the entrance of exotic species into local medical systems necessarily implies a process of acculturation. From a systems perspective, it is reasonable to infer that the process of acculturation would occur when the local medical system lost resilience and altered its stability domain [49]. Considering, however, the idea of resilience in its procedural interpretation [50], the entrance of exotic species would not lead to changes in the stability domain as long as the processes governing the local medical system remained the same.

This seems to be the case of Morrão de Cima, considering that native and exotic species are governed by the same mechanisms related to a cost-benefit rationale. Although the information about exotic plants commonly comes from outside the community, once inside the system, it behaves very similarly to native plants. This is because they enter the local networks of cultural transmission, are subject to experimentation and internal validation. Furthermore, as with native plants, people in the community are actively involved in making decisions about using exotic species, which differs from an allopathic-based medical system, whose knowledge about structure and functioning is often restricted to physicians and other health care professionals (official health).

Thus, the processes that govern the medical system (cultural transmission, validation, experimentation and active involvement in the decision of use) do not seem to be altered by the growing importance of exotic plants. However, in order to draw more convincing conclusions, it is necessary to thoroughly investigate the processes of cultural transmission and experimentation involving exotic plants. A good start could be the identification of recently introduced plants in medical systems, which would function as models for understanding the behavior of exotic plants in such systems. For the community of Morrão de Cima, we could identify two recently introduced species, both used to treat cancer: *Morinda citrifolia* L. (noni), which was introduced in the community about five years ago—according to local dwellers—and *Euphorbia tirucalli* L. (avelós), which was introduced about 10 years ago. Investigating the history of these species in the community (e.g. who brought them, whether they were experimented for other purposes and how knowledge was spread in the community) may help eliciting whether processes of cultural transmission are the same as those that drive native species.

The data also show that considering the people’s perception of resources is important to understand the adaptive strategies adopted in the interactions with the environment. The manner people perceive the costs and benefits in resource uses can provide explanations for understanding people’s decision-making in choosing one resource for use over another in the treatment of diseases. In this sense, these data provide implications for the construction of future formal ecological models that consider human behavior for the understanding of human adaptations to their environments, a necessity pointed out by some authors [8, 9]. From the theoretical point of view, in future models cost and benefit variables perceived by the people in the acquisition of different resources can be inserted in order to predict behaviors of selection and use of resources.

## Conclusions

This study contributes to the complexification of the role of exotic species in medical systems beyond the attribution of acculturation. Thus, in order to avoid the loss of knowledge regarding native plants and to guarantee biocultural conservation, it is necessary to create strategies to amplify the advantages of these species, such as the incentive to plant them, which can increase their availability, bringing the native species closer to people.

Finally, it is necessary to extend the study to other therapeutic indications in order to understand the behavior of the medical system in a more comprehensive way. It is also necessary to extend this research to other socio-environmental contexts, such as crop restriction scenarios (e.g. regions with severe water restrictions), scenarios in which agriculture does not play a central role (e.g. hunter/gatherer societies), among others. In these cases, the adaptive advantages would be expected to be shifted to the native species.

## Supporting information

S1 Dataset Database.xlsx. (XLSX)
